# Selective Adsorption and Separation of Proteins by Ligand-Modified Nanofiber Fabric

**DOI:** 10.3390/polym13142313

**Published:** 2021-07-14

**Authors:** Song Liu, Yasuhito Mukai

**Affiliations:** Department of Chemical Systems Engineering, Nagoya University, Furo-cho, Chikusa-ku, Nagoya 4648603, Japan; liusong@morimatsu.cn

**Keywords:** nanofiber fabric, protein, affinity adsorption, selective separation

## Abstract

Electrospun polyvinyl alcohol (PVA) nanofiber fabric was modified by Cibacron Blue F3GA (CB) to enhance the affinity of the fabric. Batch experiments were performed to study the nanofiber fabric’s bovine hemoglobin (BHb) adsorption capacity at different protein concentrations before and after modification. The maximum BHb adsorption capacity of the modified nanofiber fabric was 686 mg/g, which was much larger than the 58 mg/g of the original fabric. After that, the effect of feed concentration and permeation rate on the dynamic adsorption behaviors for BHb of the nanofiber fabric was investigated. The pH impact on BHb and bovine serum albumin (BSA) adsorption was examined by static adsorption experiments of single protein solutions. The selective separation experiments of the BHb–BSA binary solution were carried out at the optimal pH value, and a high selectivity factor of 5.45 for BHb was achieved. Finally, the reusability of the nanofiber fabric was examined using three adsorption–elution cycle tests. This research demonstrated the potential of the CB-modified PVA nanofiber fabric in protein adsorption and selective separation.

## 1. Introduction

Proteins are biological macromolecules that maintain the integrity of the cell structure and function [1]. Proteins are abundant in biology and are essential building blocks of life [2]. All metabolic activities of living organisms, including growth, development and reproduction, are related to the activity and metabolism of proteins. The cells of living organisms consist of different kinds of proteins, each of which has a different structure and function [3,4,5]. Proteins generally exist in complex mixed forms in tissues and cells, and various types of cells contain thousands of different proteins. As proteins vary in size, charge and water solubility, it is challenging to obtain target proteins from mixtures [6,7]. Protein separation and purification have a wide range of applications in our daily life, such as dairy [8], food industry [9], biomedicine [10], etc. The overall goal of protein isolation and purification is to increase the purity of the product and to maintain the isolated protein’s biological activity. It is desirable to achieve high production efficiency and high product quality while reducing the cost of the isolation process.

Precipitation, filtration, dialysis and chromatography techniques have been utilized for industrial separations [11,12,13,14]. Filtration is one of the most broadly used processes in protein separation. Filtration separation is based on the variability of the size and shape of protein molecules [15], where the protein solution is driven by a pressure difference to flow through the porous filter media. Protein molecules for which the sizes are smaller than the filter pore size pass through the filter medium, while protein molecules of larger sizes are retained, resulting in the separation and purification of proteins [16,17]. Taking the widely used ultrafiltration membrane as an example, the mass transfer resistance of proteins through the ultrafiltration membrane is large, which leads to high resistance. During the ultrafiltration process, pore blockage and fouling usually lead to a gradual decrease in treatment flux [18]. Furthermore, conventional ultrafiltration is limited to separating solutes with molecular size differences of more than 10 times [19]. The separation selectivity is hardly controllable for protein molecules of similar sizes [20].

The tedious process of biological product recovery dramatically impacts the final product cost because separation and purification account for 70% to 80% of the total biological process cost [21]. The significant growth in the protein market poses a considerable challenge to industrial production, particularly downstream processing, due to its limited production capacities and high cost. It is necessary to develop simple, rapid, scalable and economically feasible methods for protein separation. Polymer nanomaterials technology has brought a profound impact on the fields of artificial intelligence [22], telecommunication [23] and energy [24] and has provided a direction for the solution to the troubles in protein separation. Electrospun nanofibers are characterized by large specific surface ratios, high porosity (over 80%), superior mechanical strength, excellent controllability of the spinning process and a wide variety of raw materials [25,26,27]. It has attracted great attention from researchers in the field of adsorption and separation [28,29,30].

Up until now, a series of electrospun nanofibers has been successfully prepared for the adsorption and separation of protein [31,32,33]. Much smaller fiber diameters and increased surface areas are accessible through the electrospinning technique compared with currently available textile fibers [34]. Additionally, the large surface area provides abundant sites for protein adsorption. Electrospun nanofibers are ideal materials for a protein affinity medium because of their adjustable functionality and open porous structures. After modification by affinity ligands, the resulting nanofiber fabrics can capture target protein molecules based on highly specific binding interactions rather than on molecular weight, size or charge. Duan et al. [35] fabricated a novel metal-chelating affinity nanofiber fabric by methodically modifying the cellulose nanofiber fabric with an intermediate bridging agent of cyanuric chloride, chelating the agent of iminodiacetic acid (IDA) and affinity ligand of Fe^3+^ ions. The prepared Fe^3+^ ions’ immobilized nanofiber fabric presented a high lysozyme adsorption capacity of 365 mg/g. Reactive dyes can interact with the active sites of many proteins by mimicking the structure of the substrate or cofactors of these proteins and are excellent ligand candidates for affinity [36]. Ng et al. [37] developed a chitosan and Reactive Orange 4 modified polyacrylonitrile nanofiber fabric. The obtained dye affinity fabric was used to separate the lysozyme from chicken egg whites, and the total recovery ratio of lysozyme was up to 98.6%, with a purification multiple of 56.9 times. In two recent reports from our laboratory [38,39], we enhanced the affinity of the polyvinyl alcohol (PVA) nanofiber fabrics using Cibacron Blue F3GA (CB), which is a dye with specificity and binding abilities to a series of proteins. Then, the static and dynamic adsorption and desorption behaviors of the PVA nanofiber fabrics for bovine serum albumin (BSA) were investigated. The CB-enhanced PVA nanofiber fabric had a BSA adsorption capacity of 769 mg/g. To continue the research on protein separation, we are committed to the separation of proteins with similar sizes by nanofiber fabric. This research is not limited to the adsorption of a single protein and is more informative about the separation process of protein mixtures.

In this research, bovine hemoglobin (BHb) and BSA were used as model proteins for selective separation. Hemoglobin is a carrier of oxygen and aids the transport of carbon dioxide, and BHb has more than 85% amino acid sequence homology with human hemoglobin [40]. Serum albumin maintains the osmotic pressure and pH of the blood and transports a wide variety of endogenous and exogenous compounds, and BSA displays approximately 76% sequence homology with human serum albumin [41]. The molecular weights of BHb and BSA are 64,500 and 67,000, respectively [42,43]. The sizes of BHb and BSA are too close to be effectively separated by conventional filtration operations. First, the PVA nanofiber fabric was modified with CB to improve its affinity. Next, the static and dynamic adsorption performances of the PVA nanofiber fabrics for BHb before and after the CB modification were investigated. Then, the effect of pH on the BHb and BSA adsorption performance was examined. Finally, the selective separation experiments of BHb and BSA were carried out at the optimal pH value, with the reusability of the PVA nanofiber fabrics also being studied.

## 2. Materials and Methods

### 2.1. Materials

Electrospun polyvinyl alcohol (PVA) nanofiber fabrics were obtained from Japan Vilene Company, Ltd., Koga, Japan. The molecular weight of the PVA is 66,000–79,000, and the mass per unit area of the PVA nanofiber fabric is 40 g/m^2^. Cibacron Blue F3GA (CB) was supplied by Polysciences, Inc., Tokyo, Japan. Bovine hemoglobin (BHb) and bovine serum albumin (BSA) were purchased from Sigma-Aldrich Co. LLC., Tokyo, Japan. The other chemicals were supplied by Wako Pure Chemical Industries, Ltd., Osaka, Japan.

### 2.2. Preparation of CB-Modified PVA Nanofiber Fabric

The CB-modified PVA nanofiber fabrics were prepared according to previously reported methods [38,44,45]. One hundred micrograms of CB was dissolved in 10 mL of water to prepare a dyeing solution. Then, the dyeing solution was heated to 60 °C, and a PVA nanofiber fabric was soaked in the solution for 30 min. After that, 2 g of NaCl was added to stimulate the deposition of the dye on the internal surface of the PVA nanofiber fabric. After maintaining the fabric at 60 °C for 1 h, 0.2 g of Na_2_CO_3_ sodium carbonate was added to adjust the solution’s pH value to accelerate the reaction between the dye and the PVA nanofiber fabric, which took place at 80 °C for 2 h. Finally, the dyed PVA nanofiber fabric was thoroughly washed with deionized water until the absence of CB molecules in the wash was detected by measuring the UV-vis absorbance at 600 nm (the maximum absorption wavelength of CB).

### 2.3. BHb Adsorption Studies

The BHb adsorption capacities of the original and CB-modified PVA nanofiber fabrics were measured under different initial BHb concentrations at pH = 6.8 (BHb’s isoelectric point). The two types of fabrics were immersed into an 8 mL BHb solution with concentrations varying from 1 g/L to 6 g/L. The static adsorption experiments were carried out at 25 °C for 6 h. The BHb solution concentrations were detected by a UV-vis spectrophotometer at 406 nm before and after adsorption. To analyze the adsorption results, the amount of adsorbed protein per unit mass of fabric was determined with the following Equation:

(1) $$q=\frac{\left(C_{0}-C\right)V}{W}$$

where *q* is the adsorption capacity (mg/g); *C*_0_ and *C* are the initial and the final BHb concentrations (mg/L), respectively; *V* is the volume of the solution (L); and *W* is the fiber mass (g). The BHb adsorption capacities were plotted against the final concentrations to obtain an adsorption curve.

**Figure 1:**
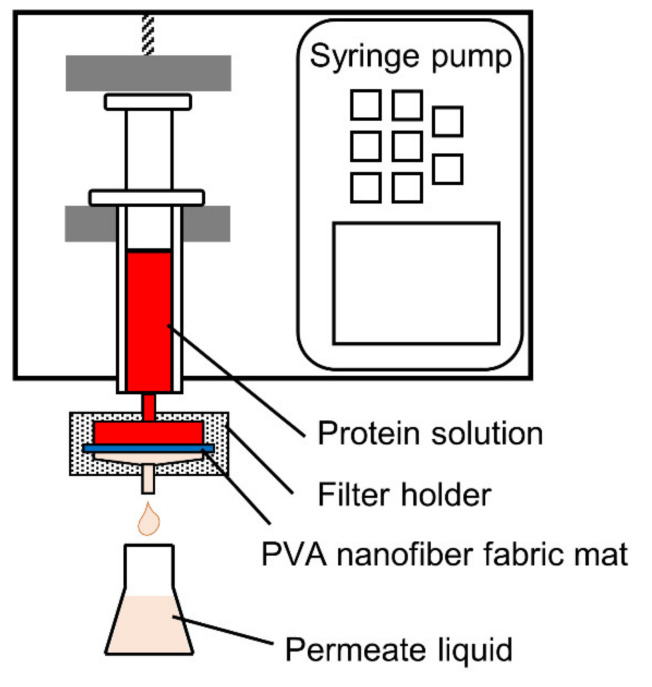
Schematic diagram of the dynamic adsorption apparatus.

The dynamic adsorption performance of BHb on the PVA nanofiber fabrics was studied by permeation tests. Figure 1 depicts the experimental setup. The PVA nanofiber fabric with a weight of 19.6 mg was placed in the Millipore filter holder (SX0002500) with an effective diameter of 2.2 cm and a permeation area of 3.8 cm^2^. Then, the inlet of the filter holder was mounted to the outlet of the syringe by a threaded connection. Subsequently, a syringe pump (SRS-2, AS ONE Co., Osaka, Japan) was used to inject the BHb solution into the filter holder. After setting the flow rate, the permeation experiment was started by collecting the permeate from the outlet below the filter holder and by monitoring the concentration of BHb. Detailed studies were conducted at 25 °C, BHb feed concentrations of 0.3–1.2 g/L and permeation rates of 2–10 mL/h. The momentary rejection ratio *R_m_* for protein was determined according to the following Equation:

(2) $$R_{m}=1-\frac{C_{p}}{C_{0}}$$

where *C*_0_ (g/L) and *C_p_* (g/L) are the protein concentrations in the feed solution and the permeate, respectively.

At a cumulative permeate volume of *V* (mL), the rejection ratio *R_c_* for protein of the CB modified PVA nanofiber fabric is given by

(3) $$R_{c}=\frac{1}{V}\int_{0}^{V}R_{m}dV$$

### 2.4. Selective Separation of Binary BHb–BSA Solution

The single-protein adsorption experiments were carried out at 25 °C for 6 h. The single-component concentrations of BHb or BSA solution were 3 g/L. The effect of pH on the adsorption capacity was investigated using different pH values ranging from 4 to 9. The protein concentrations before and after adsorption were measured using a UV-vis spectrometer, referring to the absorption band at 406 nm for BHb and 280 nm for BSA.

Selective experiments were carried out with the permeation apparatus at the optimum pH of 6.8. The initial concentrations of both proteins were 0.6 g/L in the BHb–BSA binary solution. The BHb solution exhibits two characteristic absorbance peaks at 280 nm and 406 nm, while the absorbance of the BSA solution exhibits a maximum around 280 nm and can be negligible around 406 nm. Accordingly, the BHb concentration was determined directly from the absorbance at 406 nm. The BSA concentration was determined at 280 nm by subtracting the contribution of BHb from the concentration detected at 406 nm. The rejection ratio for BHb or BSA is calculated according to Equation (3), and the selectivity factor *S* of protein is defined by the following equation:

(4) $$S=\frac{R_{c,BHb}}{R_{c,BSA}}$$

Finally, the reusability tests for BHB–BSA binary protein dynamic adsorption were repeated for three cycles to examine the stability of the CB-modified PVA nanofiber fabrics.

## 3. Results and Discussion

### 3.1. Characteristics of the PVA Nanofiber Fabrics

**Figure 2:**
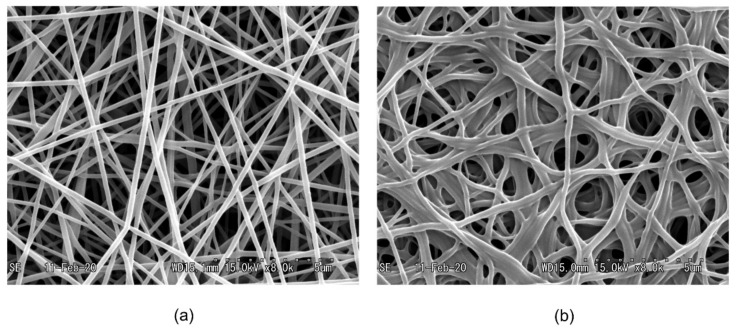
SEM images of (**a**) the original PVA nanofiber fabrics and (**b**) the CB-modified PVA nanofiber fabrics.

Morphologies of the PVA nanofiber fabrics before and after CB modification were observed by scanning electron microscopy (SEM, S4300, Hitachi High-Technologies Corporation, Tokyo, Japan), operating at 15 kV. Figure 2 shows the SEM images of the PVA nanofiber fabrics. The fiber diameters were analyzed using the ImageJ software. The original PVA nanofiber fabrics were smoother than the CB-modified PVA nanofiber fabrics. After the CB modification, the average fiber diameter increased from the original 224 nm to 238 nm. The CB molecules were covalently immobilized on the fabric surface, which is one of the reasons for the coarsening of the fibers. In addition, the CB modification treatment was carried out in solution, and the hydrophilic PVA nanofiber fabrics probably had a slight swelling.

### 3.2. Static Adsorption Isotherm of BHb

**Figure 3:**
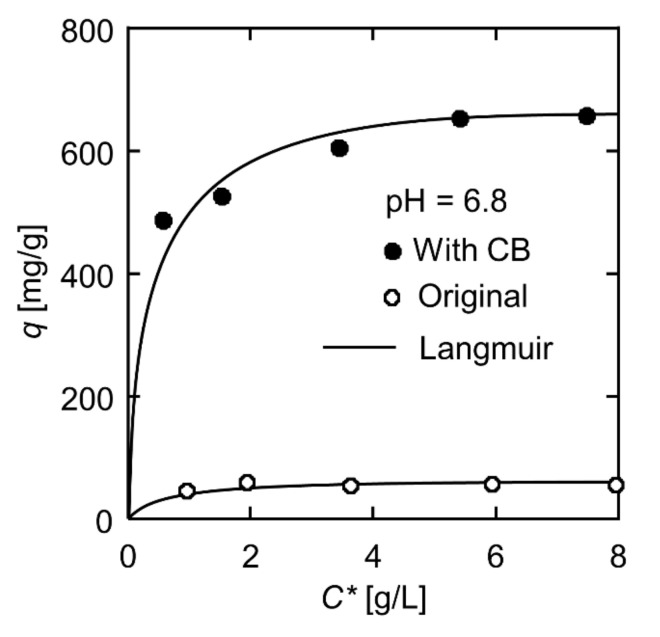
Effect of the BHb initial concentration on adsorption capacities of the original and CB-modified PVA nanofiber fabrics.

In order to evaluate the BHb adsorption capacity of the PVA nanofiber fabrics before and after CB modification, the effect of the initial concentration on the adsorption capacity of the nanofiber fabrics was studied, with the results shown in Figure 3.

The BHb adsorption on the PVA nanofiber fabrics increased with the increase in the BHb concentration. The adsorption capacity of the CB-modified PVA nanofiber fabrics was markedly greater than that of the original fabrics. The adsorption amount of BHb on the original PVA nanofiber fabrics was at a low level in all experimental concentrations, while the adsorption amount of the modified nanofiber fabrics was around 655 mg/g when the BHb concentration exceeded 6.0 g/L. The excellent adsorption capacity of the modified nanofiber fabrics is attributed to the affinity of CB molecules. The interaction between the CB molecule and BHb is a consequence of the combined effects of electrostatic and hydrophobic interactions, and hydrogen bonding. As a monochlorotriazine dye, the CB molecule contains three sulfonic acid groups, and four basic primary and secondary amino groups. The triazine part of CB is used to fix onto the PVA matrix, while the three sulfonic acid groups and the multiple aromatic parts are mainly responsible for the CB–BHb binding [46].

The Langmuir adsorption isotherm is one of the most widely used adsorption isotherms, which is used to fit adsorption equilibrium data. It is assumed that adsorption is a monolayer type occurring on the homogeneous surface of the adsorbent [47,48]. The linear form of the Langmuir equation is expressed as follows:

(5) $$\frac{C^{*}}{q_{e}}=\frac{C^{*}}{q_{s}}+\frac{1}{Kq_{s}}$$

where *q_s_* (mg/g) is the maximum adsorption capacity, *q_e_* (mg/g) is the equilibrium adsorption capacity, C* (g/L) is the equilibrium BHb concentration and *K* (L/g) is the dissociation constant of the system.

By fitting the experimental data to the Langmuir linear equation, the maximum adsorption capacities of BHb on the PVA nanofiber fabrics before and after CB modification were calculated to be 58 mg/g and 686 mg/g, respectively. Zhang et al. [49] prepared magnetic carbon nanotubes with a hierarchical copper silicate nanostructure for BHb adsorption, with the maximum BHb adsorption capacity being 302 mg/g. Wang et al. [50] reported that magnetic mesoporous ytterbium silicate microspheres achieved a BHb adsorption capacity of 304 mg/g. Compared to these materials, the CB-modified PVA nanofiber fabric has a distinct advantage in adsorption capacity.

### 3.3. BHb Dynamic Adsorption Performance

Dynamic adsorption operation offers the benefits of automatic control, labor saving and easy integration with other continuous processes. Thus, we tested the BHb dynamic adsorption behavior of the CB-modified PVA nanofiber fabrics.

**Figure 4:**
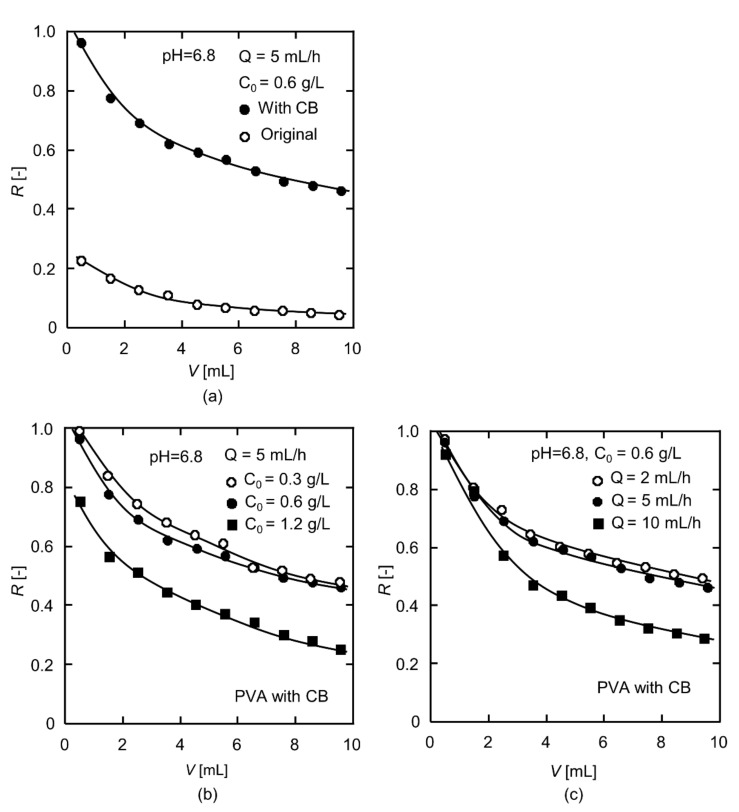
Dynamic adsorption test results for (**a**) the original and CB-modified PVA nanofiber fabrics, (**b**) the effect of feed concentrations on the CB-modified PVA nanofiber fabrics, and (**c**) the effect of permeation rates on the CB-modified PVA nanofiber fabrics.

Figure 4a shows the changes in the BHb rejection ratio over the permeation volume from 0 to 10 mL for both the original and CB-modified PVA nanofiber fabrics. The BHb rejection ratio by both nanofiber fabrics showed a downward trend with the increase in permeation volume. Whereas the BHb rejection ratio of the CB PVA nanofiber-modified nanofiber fabric remained at a high level, reducing from 1.0 to 0.62, the BHb rejection ratio of the original nanofiber fabric was meagre, being 0.23 at the beginning and only 0.10 at the end of the experiment. The enhancement in the protein rejection ratio of the CB-modified PVA nanofiber fabric comes from the affinity effect of CB molecules.

Figure 4b presents the BHb rejection ratio of the CB-modified PVA nanofiber fabrics varied with permeation volume at different feed concentrations. For the highest feed concentration of 1.2 g/L, the contact probability for interaction between BHb molecules in the mobile phase and nanofiber surface was the highest, and the driving force was the largest. As a result, the BHb adsorption sites on the nanofiber surface were occupied sooner and the rejection ratio was markedly lower than that of the other two dilute solutions. For the solutions with concentrations of 0.3 and 0.6 g/L, the changes in the BHb rejection ratio were similar, but the rejection ratio values were higher at 0.3 g/L.

The influence of permeation rate on the BHb rejection ratio was examined, and the results are shown in Figure 4c. When the permeation rate was 10 mL/h, the BHb rejection ratio was dramatically reduced due to the insufficient retention time of BHb molecules in the pores of the nanofiber fabric. In contrast, the BHb rejection ratio decreased more slowly in the permeation experiments, with permeation rates of 2 and 5 mL/h. Therefore, it is necessary to allow for sufficient residence time in order to obtain the protein rejection effect. Additionally, attention needs to be paid to the balance between the productivity and rejection ratio during the dynamic adsorption operation.

### 3.4. Effect of pH on BHb and BSA Adsorption

Both BHb and BSA are homologous proteins derived from bovine blood, and their molecular weights are so close that they are difficult to separate with conventional filtration methods. Nevertheless, it is worth noting that the two proteins have different isoelectric points, 5.0 and 6.8, respectively. Proteins are amphoteric biomolecules because the amino acids that make up proteins contain both basic and acidic functional groups [43]. The protein surface charge depends on the pH value of the protein solution. At the isoelectric point, the electric charge of protein becomes zero. When the pH value of the protein solution is lower than the isoelectric point, the protein surface has a positive charge; in the contrary situation, the protein is negatively charged. Therefore, pH has a considerable influence on the adsorption capacity.

**Figure 5:**
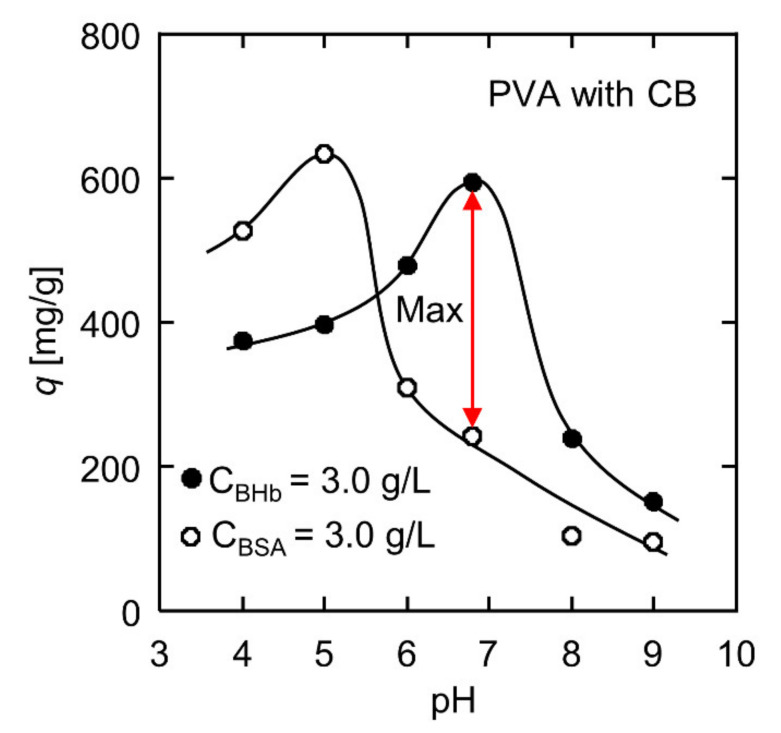
Effect of pH on BHb and BSA adsorption performances.

The pH effect on the adsorption capacity in a single component solution of BHb or BSA was studied by the static adsorption experiments. As BSA undergoes irreversible structural transitions in the range of pH < 4 [51], the experiments were performed at pH = 4–9. Figure 5 displays the adsorption amounts of the CB-modified PVA nanofiber fabrics towards BHb and BSA. Under different pH values, the adsorption capacities of BHb and BSA were different. The greater the difference between the adsorption amounts, the more likely it is to realize selective separation. The adsorption capacity of BHb was the maximum at its corresponding isoelectric point of 6.8. The BHb adsorption capacity decreased in varying degrees at other pH values. Similarly, the BSA adsorption capacity obtained its maximum at an isoelectric point of 5.0. At the isoelectric point, the repulsive force between protein molecules was minimal because the protein charge was zero. As a result, more protein molecules were arranged on the nanofiber surface during the affinity interaction with the CB molecules immobilized on the nanofiber fabrics. At pH = 6.8, the adsorption amounts of the CB-modified PVA nanofiber fabrics were 594 mg/g for BHb and 244 mg/g for BSA. The difference in the adsorption amounts was more extensive than that of other pH values, about 350 mg/g. In this case, pH = 6.8 was taken as the condition of the binary protein separation.

### 3.5. Selective Separation of Binary BHb–BSA Solution

**Figure 6:**
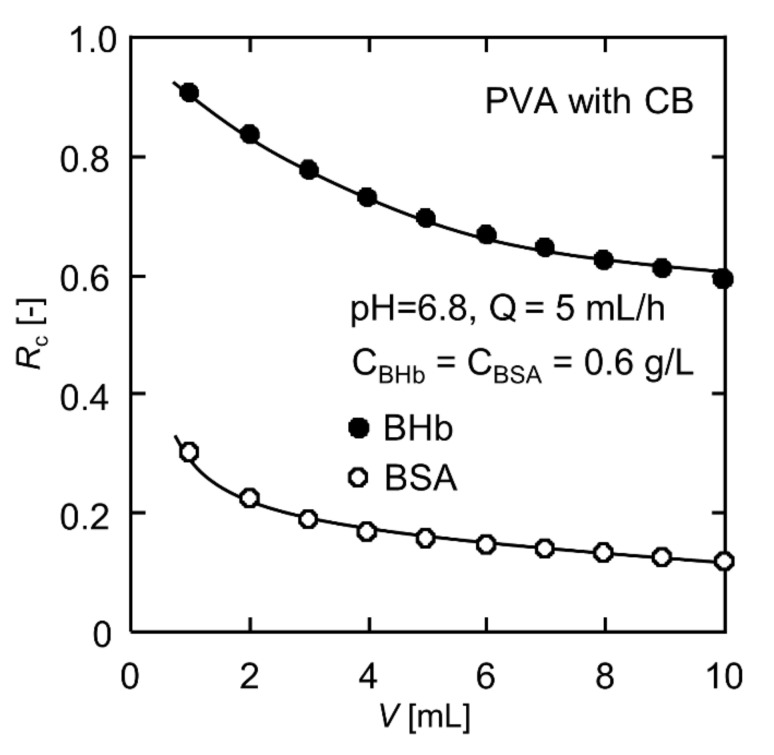
Selective separation results for the BHb–BSA binary solution.

Dynamic adsorption experiments of the CB-modified PVA nanofiber fabrics were conducted with binary BHb–BSA solutions. Figure 6 shows the variation in the rejection ratios for BHb and BSA versus the permeation volume. From the beginning of the permeation experiment, the rejection ratio for BHb was much higher than BSA, and the rejection ratios decreased gradually as the permeation volume increased. The selectivity factor *S* of BHb for the initial 1 mL of the permeate was about 3.01. The initial *S* seemed unsatisfactory due to a large number of vacant active sites on the nanofiber surface where BHb and BSA molecules were both able to attach at the beginning of the experiment. Similarly, a higher mass per unit area of the PVA nanofiber fabrics does not necessarily lead to an improvement in selectivity factor because the rejection ratio of both BHb and BSA will be maintained at a high level due to abundant adsorption sites. However, at an end permeation volume of 10 mL, the rejection ratios for BHb and BSA were 0.60 and 0.11, respectively, and *S* rose to 5.45. The dynamic adsorption operation was carried out only once, and it showed a good separation possibility. Higher selectivity factors are expected in multi-stage continuous dynamic adsorption operations, which needs to be investigated in future work.

The BHb molecules with zero charge occupied more active sites on the nanofiber surface. On the contrary, at pH = 6.8, BSA was negatively charged and had difficulty in close arrangements on the nanofiber surface due to the electrostatic repulsion between molecules, resulting in a weaker competition for active sites on the nanofiber surface compared to BHb. In addition, the size of the BHb molecule is 6.4 nm × 5.5 nm × 5 nm, with a spherical shape, while the size of the BSA molecule is 14 nm × 3.8 nm × 3.8 nm, with a shape similar to a prolate ellipsoid [52]. When BSA molecules flowed through the intricate pores inside the nanofiber fabrics, in order to obtain minimal interaction with the nanofiber surface, the ellipsoidal molecules might align their long axis parallel to the centerline of the nanofiber, resulting in a lower hydrodynamic hindrance than that of BHb molecules. In contrast, spherical BHb molecules were more easily attracted by the CB molecules immobilized on the nanofiber surface when passing through the internal pores of the nanofiber fabric.

### 3.6. Desorption and Reusability

It has been reported that increasing the solution ionic strength leads to a decrease in protein adsorption amount [38,53]. Salts in the adsorption medium can lead to coordination of the deprotonated sulfonic acid groups of CB molecule with sodium ions; on the other hand, the distortion of existing salt bridges contributes to low protein adsorption at high ionic strength [45]. In addition, moving the pH of the adsorption medium away from the protein’s isoelectric point is also detrimental to adsorption. Thus, a phosphate buffer at pH = 10.0 containing 1.0 M NaCl was used as the eluent to desorb BHb and BSA from the protein adsorbed CB-modified PVA nanofiber fabrics. According to our previous analysis of protein concentrations at different pH values, there was no significant difference in absorbance at pH = 10 for the same concentration of protein solution while the absorbance severely decayed at pH ≤ 3. Therefore, BHb and BSA are more sensitive to acidity than alkalinity, and the eluent of pH = 10 does not cause structural damage to proteins. The protein-adsorbed nanofiber fabric was eluted with 20 mL eluent at a rate of 5 mL/h using the permeation apparatus, and more than 96% of the protein molecules were eluted off. The eluent can be desalinated by an ultrafiltration operation after pH adjustment, and then, the desorbed BHb and BSA can be concentrated for further recovery.

**Figure 7:**
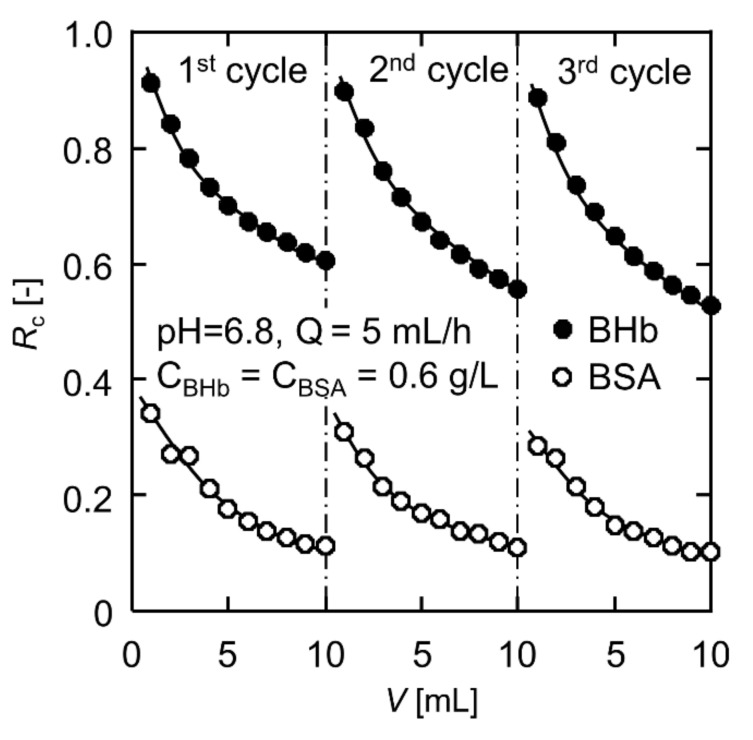
Reusability test of the CB-modified PVA nanofiber fabric for selective separation.

The adsorption–desorption was repeated for three cycles to evaluate the reusability of the CB-modified PVA nanofiber fabrics. Figure 7 displays the changes in rejection ratios for BHb and BSA with the permeation volume during the three adsorption cycles. The rejection ratios for both proteins declined slightly. However, the selectivity factor *S* maintained above 5 at the end permeate volume of 10 mL in all three cycles, with 5.36, 5.07 and 5.13, respectively. It is demonstrated that the CB-modified PVA nanofiber fabrics can be reused without losing activity easily.

## 4. Conclusions

In this research, CB-modified PVA nanofiber fabrics were fabricated via immobilization of CB molecules on the nanofiber surface. The batch experiments showed that the modified nanofiber fabrics have a high adsorption capacity for BHb. The Langmuir isotherm was used to fit the experimental data, and the maximum adsorption capacity of the nanofiber fabric was calculated to be 686 mg/g. Then, dynamic experiments were performed to determine the effect of feed concentration and permeation rate on the BHb rejection ratio. In the single-protein static adsorption experiments, the pH impact on the adsorption of BHb and BSA was investigated separately, where the pH value with the largest difference in the adsorption amount was explored. Finally, a high selectivity factor of 5.45 for BHb was achieved during the dynamic adsorption of the BHb–BSA binary solution at pH = 6.8, corresponding to the isoelectric point of BHb. The selectivity factors for BHb were maintained above 5 in the three adsorption–elution cycles, indicating the reusability of the nanofiber fabric. This work demonstrated the potential of the CB-modified PVA nanofiber fabric in the selective separation for proteins with similar sizes.

## Data Availability

The data presented in this study are available from the corresponding author upon request.
